# Exploring CCND1 as a Key Target of *Acorus calamus* Against RSV Infection: Network Pharmacology, Molecular Docking, and Bioinformatics Analysis

**DOI:** 10.3390/cimb47090695

**Published:** 2025-08-27

**Authors:** Haojing Chang, Li Shao, Ke Tao, Xiangjun Chen, Hehe Liao, Wang Liao, Bei Xue, Shaokang Wang

**Affiliations:** 1 Key Laboratory of Environmental Medicine and Engineering of Ministry of Education, Department of Nutrition and Food Hygiene, School of Public Health, Southeast University, Nanjing 210009, China; chj@seu.edu.cn (H.C.); shaoli@xzmu.edu.cn (L.S.); augpeche20@163.com (K.T.); xjchen@xzmu.edu.cn (X.C.); wangliao@seu.edu.cn (W.L.); 2 Clinical Medical Research Center for Plateau Gastroenterological Disease of Xizang Autonomous Region, School of Medicine, Xizang Minzu University, Xianyang 712082, China; huhuliaoyh@foxmail.com (H.L.); bxue@xzmu.edu.cn (B.X.)

**Keywords:** respiratory syncytial virus, *Acorus calamus*, network pharmacology, molecular docking

## Abstract

*Acorus calamus*, a traditional Tibetan medicine with potential antiviral activity but undefined mechanisms, was studied for its anti-respiratory syncytial virus (RSV) mechanisms using network pharmacology and molecular docking, given RSV’s substantial disease burden and lack of specific therapies. The primary active compounds were identified and analyzed through a literature search, the PubChem database, and the SwissADME. Relevant targets were sifted through the SwissTargetPrediction platform, OMIM, and GeneCards databases. Common targets underwent enrichment analysis using Disease Ontology (DO), Gene Ontology (GO), and Kyoto Encyclopedia of Genes and Genomes (KEGG). Molecular docking and GEO datasets were used for further analysis. Among the screened data, 268 targets were associated with *Acorus calamus* compounds and 1633 with RSV. KEGG analysis of the shared targets revealed potential therapeutic roles via the PI3K–Akt and JAK–STAT signaling pathways. Molecular docking results demonstrated that CCND1, EGFR, and SRC exhibited relatively lower binding energies with compounds in comparison to other proteins, suggesting better interactions, and GEO-derived RSV datasets further validated CCND1’s significance. This study demonstrates *Acorus calamus*’s anti-RSV activity and its potential mechanism, providing a theoretical foundation for the effective active ingredients of *Acorus calamus* targeting CCND1 as a strategy to combat RSV infection.

## 1. Introduction

Respiratory syncytial virus (RSV) is the preeminent etiological agent of lower respiratory tract infections (LRTIs), particularly in the pediatric population [1]. It is the leading cause of hospital admissions for respiratory diseases among infants and children worldwide [2]. The global burden of disease due to RSV is significant, with substantial morbidity and mortality in infant and child populations. An annual total of over 3.6 million children under the age of five are hospitalized for RSV infections, and approximately 100,000 children under the age of five die from RSV infections [3]. Furthermore, findings from China’s acute respiratory infection surveillance further reveal that among children under 5 years old, the RSV positivity rate is as high as 25.7%, surpassing that of other viral pathogens and ranking first among all viral infections [4].

Despite its widespread prevalence and substantial disease burden, RSV is recognized as a preventable infection. In 2023, two new RSV vaccines for infants and children received approval [5,6], offering renewed optimism in the fight against RSV. However, evidence suggests that early onset and severe RSV infections can impinge on normal lung development, potentially culminating in long-term respiratory disorders [7,8]. Since vaccination does not confer complete protection against RSV infection and there remains a dearth of specific antiviral therapies for RSV, it is of utmost importance to identify targeted treatments. Consequently, there is an imperative need to develop effective treatment strategies for RSV, with a view to reducing the disease burden and the long-term effects of RSV infection.

Natural products represent a rich reservoir for drug development, with Tibetan medicine being an integral component [9]. Over time, Tibetan medical practitioners have established a comprehensive theoretical framework based on their extensive practical experiences [10]. *Acorus calamus* (ZangChangPu, ZCP) is a commonly used herb in Tibetan medicine and holds a significant place within this traditional medical system. Previous research has demonstrated that *Acorus calamus* can inhibit acute asthmatic reactions and is effective in treating chronic bronchitis [11]. Moreover, the traditional Tibetan medicine formula, Jiuwei Heiyao Fangwen powder, has exhibited remarkable potential in the prevention and management of Severe Acute Respiratory Syndrome (SARS), COVID-19, and Influenza Virus A (H1N1). Notably, *Acorus calamus* is one of the key ingredients in this formula [12]. Based on these findings, it can be hypothesized that *Acorus calamus* may possess value in the context of antiviral infections [13]. Nevertheless, current investigations into the antiviral properties of *Acorus calamus* remain limited, and the underlying mechanisms of its action against viruses are yet to be elucidated. Further research is warranted to explore the potential of *Acorus calamus* as an antiviral agent, which could contribute to the development of novel antiviral therapies.

In recent years, the advent and progression of network pharmacology have enabled in-depth exploration of the intricate relationships between herbal components and diseases. By comprehensively analyzing drug components, their corresponding disease-related targets, and elucidating the implicated biological processes and pathways, valuable insights into the mechanisms of drug therapy can be gleaned [14]. Against this backdrop, the present study adopted a network pharmacology-based approach to dissect the pharmacological effect network of *Acorus calamus* in the treatment of RSV infection. Concurrently, in an effort to explore the underlying mechanism of *Acorus calamus* in treating RSV at the molecular and pathway levels, this study integrated enrichment analysis, molecular docking, and dataset validation techniques. The overarching aim was to provide robust evidence for the utility of *Acorus calamus* in RSV treatment and to pioneer a novel approach for managing RSV infections. 

## 2. Materials and Methods

### 2.1. Screening for Active Compounds and Their Corresponding Targets in Acorus calamus

The constituent compounds were obtained from a literature search for common constituents of *Acorus calamus* and checked in the PubChem database (https://pubchem.ncbi.nlm.nih.gov) [accessed on 15 March 2025] to confirm the compound structures and collect the common names and SMILES. The active compounds were then subjected to screening in SwissADME (http://www.swissADME.ch) [accessed on 15 March 2025] according to the criteria that (1) indicated high GI absorption and (2) received a score of 2 or greater in the druglikeness entries. The compounds meeting these criteria were identified as active compounds. The SwissTargetPrediction platform (http://swisstargetprediction.ch) [accessed on 15 March 2025] was then utilized to predict potential targets based on structural similarity between known ligands and compounds to be tested, with “Homo sapiens” selected and the retention parameter set to probability > 0.1.

### 2.2. Gathering of RSV-Related Targets

Two databases, Online Mendelian Inheritance in Man (OMIM: https://omim.org/) [accessed on 17 March 2025] and The Human Gene Database (GeneCards: https://www.genecards.org/) [accessed on 17 March 2025], were queried to obtain potential targets associated with RSV. We finally integrated all the targets and removed duplicates to obtain the full range of RSV-related targets.

### 2.3. Shared Targets Were Determined from the Intersection of Active Compound Targets and RSV-Associated Targets

Common targets shared between RSV-related and predicted *Acorus calamus* targets were identified and visualized in a Venn diagram using Venny 2.1.0 (https://bioinfogp.cnb.csic.es/tools/venny/index.html) [accessed on 20 March 2025]. The common targets were imported into Cytoscape 3.10.2 to construct a herb–compound–target network diagram.

### 2.4. Constructing a Network to Visualize PPI

The common targets were analyzed for protein–protein interaction (PPI) analysis using the STRING database (http://string-db.org) [accessed on 20 March 2025], with parameters set to *Homo sapiens* and a confidence score threshold of 0.4 to ensure data reliability. The analysis results were subsequently imported into Cytoscape 3.10.2 for visualization, and the core targets in the network were selected using the CytoHubba plugin. The Maximum Clique Centrality (MCC) algorithm was selected for network topology analysis, and the top 15 genes were identified as the core targets for subsequent analysis.

### 2.5. DO, GO, and KEGG Enrichment Analyses

Using R software (version 4.3.3), the shared targets underwent enrichment analyses for Disease Ontology (DO), Gene Ontology (GO), and Kyoto Encyclopedia of Genes and Genomes (KEGG), applying *p*-value and *q*-value cutoffs of 0.05. The results of KEGG enrichment analysis were imported into Cytoscape 3.10.2, and the compound–target–pathway network diagram was drawn. Core targets were identified based on three essential topological parameters: Degree, Closeness Centrality (CC), and Betweenness Centrality (BC).

### 2.6. Molecular Docking Verification

Ligand structures were sourced from PubChem, while the 3D crystal structures of the targets were acquired from the Universal Protein Resource Database (UniProt: https://www.uniprot.org/) [accessed on 21 March 2025]. Both datasets underwent preliminary processing and were imported into AutoDockTools 1.5.6 for molecular docking, with interaction visualizations generated using PyMOL. Intermolecular interactions, including hydrogen bonding and hydrophobic interactions, were further analyzed using LigPlot. The molecular docking binding energy results were then visualized using a heat map drawn with R 4.3.3.

### 2.7. RSV Datasets Acquisition and Target Validation

Differential expression analysis of RSV datasets GSE105450 and GSE77087 from Gene Expression Omnibus (GEO: https://www.ncbi.nlm.nih.gov/geo/) [accessed on 23 March 2025] was conducted in R 4.3.3 (|logFC| > 1, *p* < 0.05), and results were illustrated via volcano plots and heatmaps. The Venn diagram was employed to identify the common targets among the core targets and the two RSV datasets, GSE105450 and GSE77087. The expression of the common targets was presented as box plots.

## 3. Results

### 3.1. The Candidate Targets of Acorus calamus and Their Corresponding Active Compounds

Numbered lists can be added as follows: A total of 26 active compounds were identified in *Acorus calamus* gathered from the literature search and PubChem database (Table 1) [15,16,17,18,19,20]. We initially retrieved a total of 118 components from the literature. The PubChem database determined the structures and PubChem CIDs of a total of 78 of these components. Fifty-six of the components passed the SwissADME criteria for drug-likeness. Through a search in the SwissTargetPrediction platform, 451 potential targets associated with 26 compounds of *Acorus calamus* were obtained, and with duplicates removed, this led to the identification of 268 targets (Supplementary Table S1).

### 3.2. RSV-Related Targets

**Figure 1:**
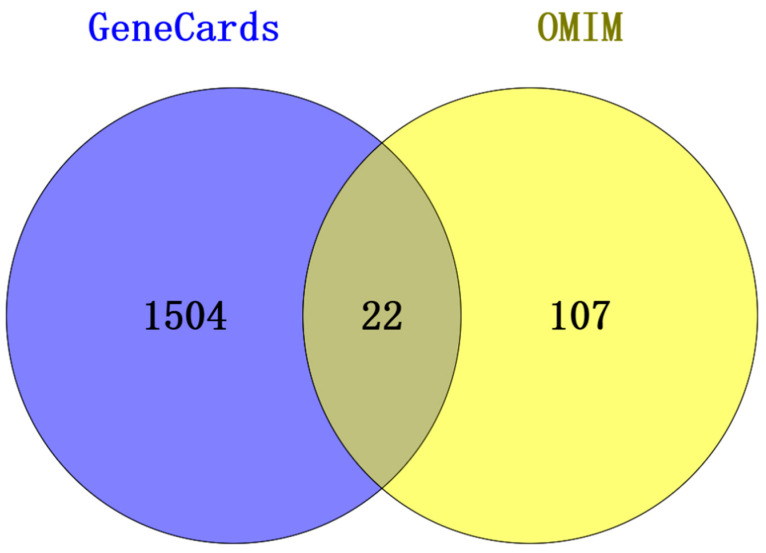
Venn diagram of 1633 common disease targets after the merger of GeneCards and OMIM.

By merging data from GeneCards and OMIM and removing overlapping entries, 1633 unique targets related to RSV were identified. A total of 1633 RSV-related targets were identified as disease targets in a Venn diagram after the merger of GeneCards and OMIM (Figure 1).

### 3.3. Construction of Herb–Compound–Target Network

**Figure 2:**
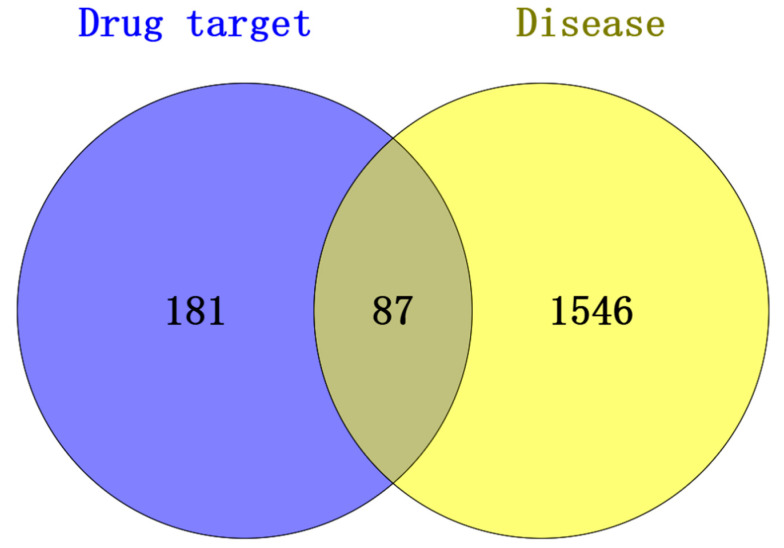
Visualization of 87 overlapping targets between *Acorus calamus* active compounds and RSV disease targets via Venn diagram.

**Figure 3:**
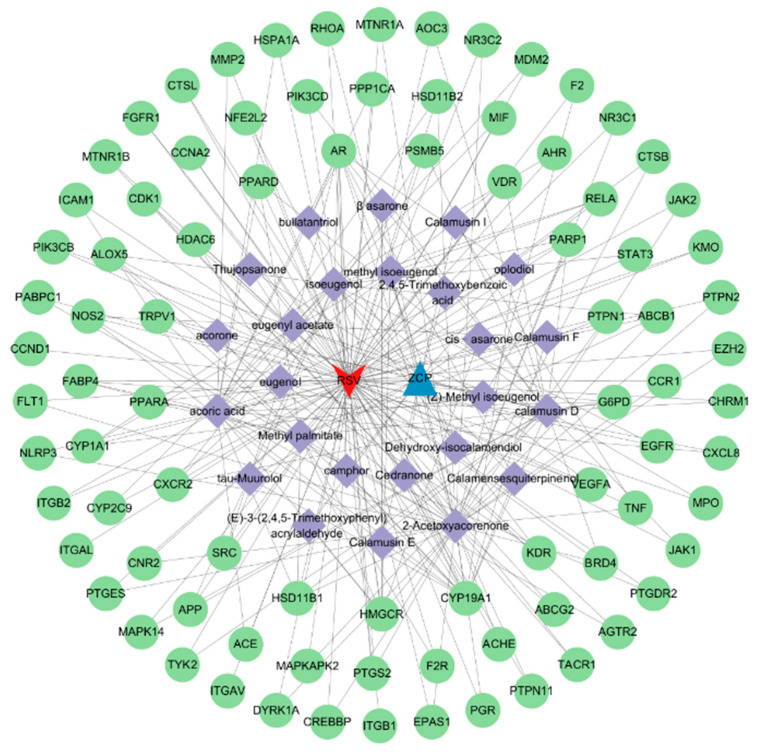
Network between herb compounds and disease targets.

Venn diagram analysis (Figure 2) revealed 87 common targets shared by the active compounds of *Acorus calamus* and RSV-associated disease targets. The details were shown in Table 2. The identified common targets may serve as potential mediators of *Acorus calamus*’s therapeutic effects against RSV. A network was constructed using Cytoscape 3.10.2, incorporating 26 active compounds and 87 associated targets (Figure 3). In this network, edges represent the interactions between compounds and their respective targets, while node size and color intensity indicate the relative strength or significance of these interactions. One component in the figure may correspond to one or more targets, indicating that the pharmaceutical composition has multi-component and multi-target properties for the treatment of disease. The top 10 compounds, sorted by degree within the herb–compound–target network, were presented in Table 3.

### 3.4. Analysis of Herb–Compound–Target Network and Construction of PPI Network for Common Targets

**Figure 4:**
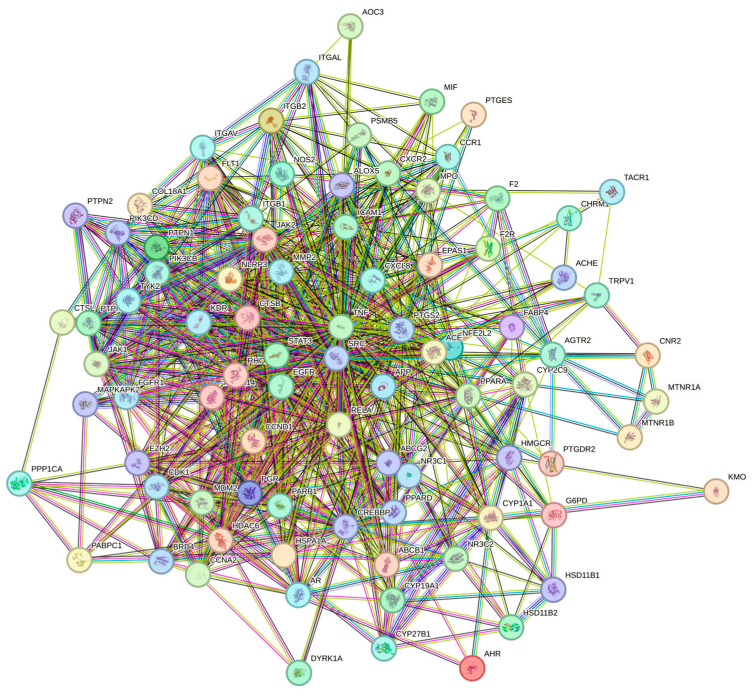
Common targets PPI network from STRING.

**Figure 5:**
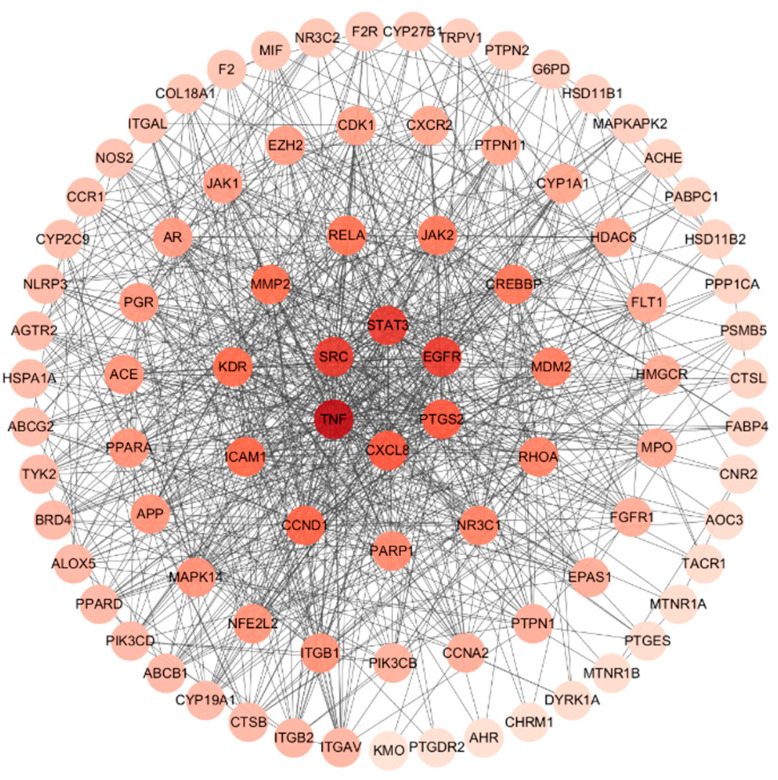
PPI network topology analysis result by Cytoscape 3.10.2.

**Figure 6:**
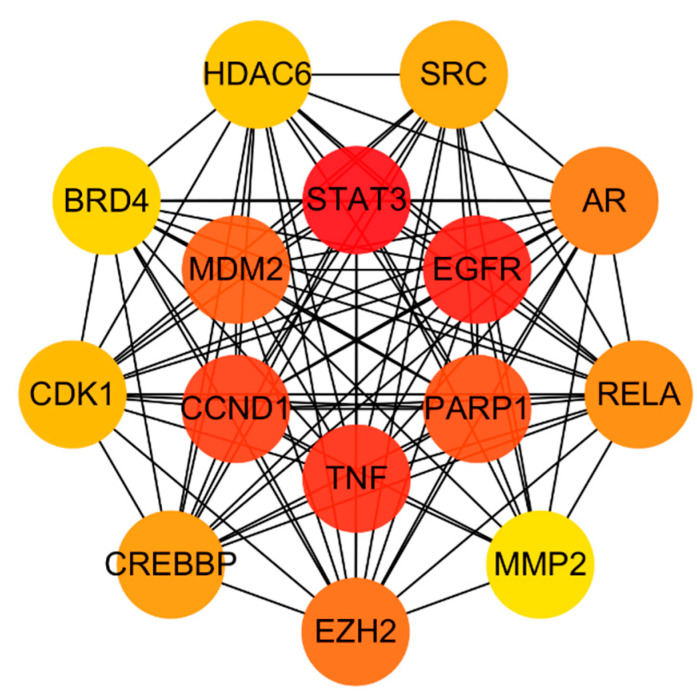
Top 15 core targets from PPI network using the CytoHubba plugin and MCC algorithm.

In order to investigate the interaction underlying *Acorus calamus* with RSV, a PPI network was constructed using STRING and Cytoscape 3.10.2, with 87 common targets forming the foundation of this network (Figure 4 and Figure 5). Color gradients of the nodes, ranging from light to dark, indicate ascending node degree values. Subsequent analysis of the network identified the top 15 genes as the core targets for further investigation (Figure 6, Table 4). This analysis employed the CytoHubba plugin and the MCC algorithm for network topology analysis.

### 3.5. DO, GO, and KEGG Enrichment Analysis

**Figure 7:**
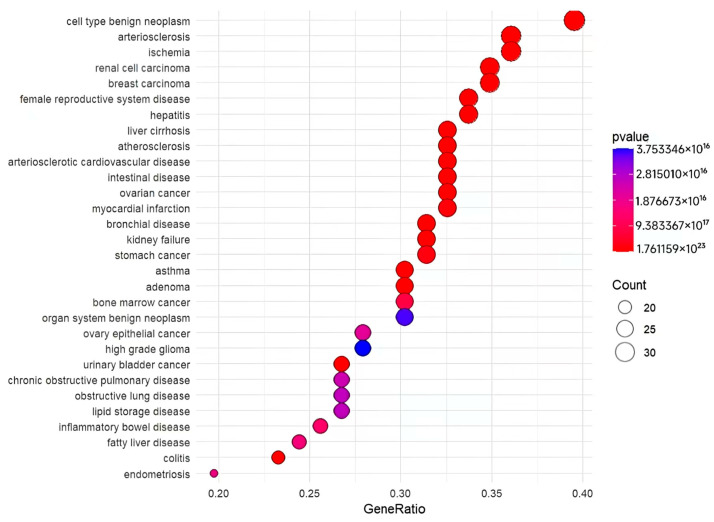
Bubble diagram illustrating the top 30 diseases identified through DO enrichment.

**Figure 8:**
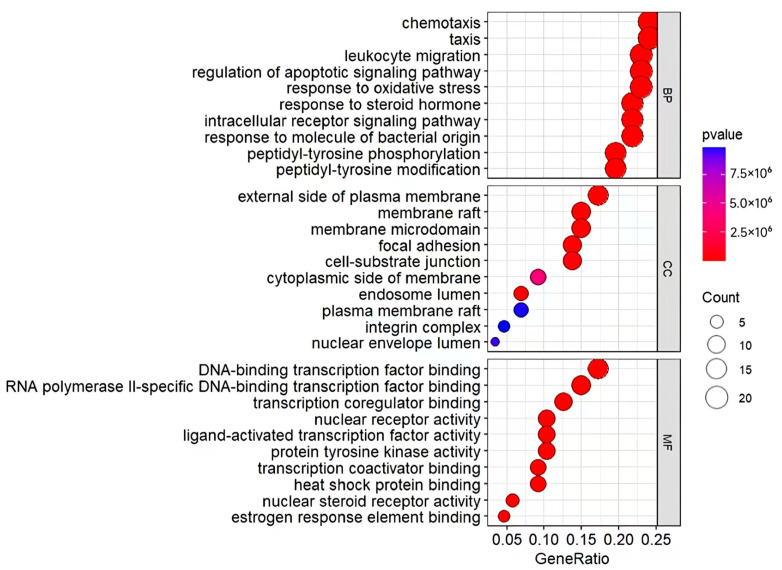
Bubble diagram representing GO functional enrichment outcomes.

**Figure 9:**
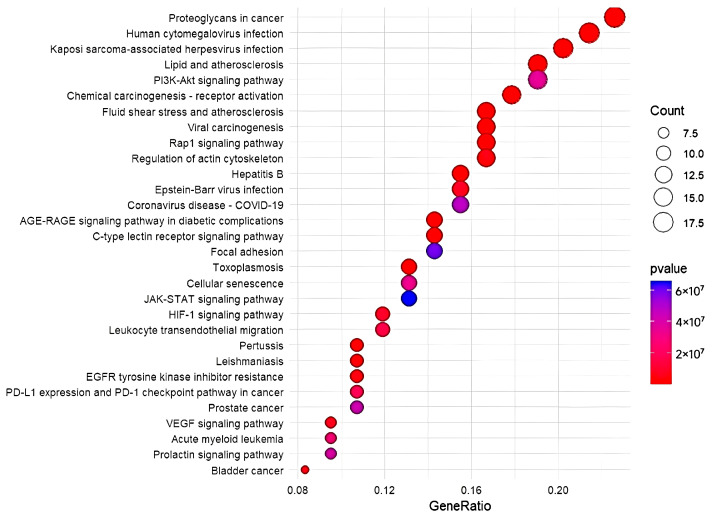
Bubble plot showing the top 30 pathways identified through KEGG enrichment analysis.

Enrichment analyses (DO, GO, and KEGG) for the 87 drug–disease-related targets were conducted in R 4.3.3, employing *p*-value and *q*-value thresholds below 0.05 for significance. A total of 696 disease terms were enriched through DO analysis. The 30 categories with the highest levels of enrichment were displayed in bubble charts. Notably, high-ranking categories such as bronchial disease may be closely related to RSV development (Figure 7). Furthermore, a GO analysis was performed, resulting in the identification of 1721 GO terms, with the allocation of 1564 to Biological Processes (BP), 43 to Cellular Components (CC), and 114 to Molecular Functions (MF) (Figure 8). The most significantly enriched BP terms were mainly associated with chemotactic response, leukocyte migration, modulation of apoptotic signaling pathways, and oxidative stress response. The CC terms predominantly focus on the external side of the plasma membrane, the membrane raft, and the membrane microdomain. Additionally, a substantial proportion of the highly enriched MF terms pertain to DNA-binding, RNA polymerase II-specific DNA-binding, and transcription coregulator binding. KEGG enrichment analysis revealed 136 distinct signaling pathways. Among the top 30 pathways, the highest-ranking, including the C-type lectin receptor, PI3K-Akt, and JAK-STAT signaling pathways, showed strong associations with RSV onset and progression (Figure 9).

### 3.6. Construction of Compound–Target–Pathway Network and Identification of Core Targets for RSV

**Figure 10:**
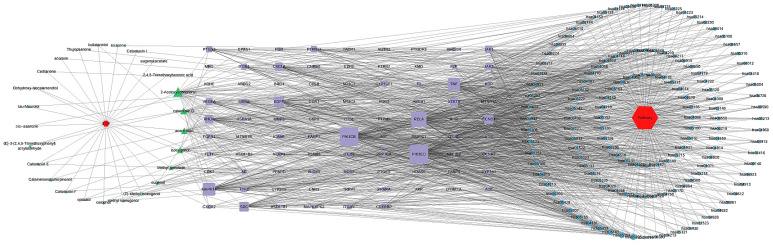
Compound–target–pathway network.

**Figure 11:**
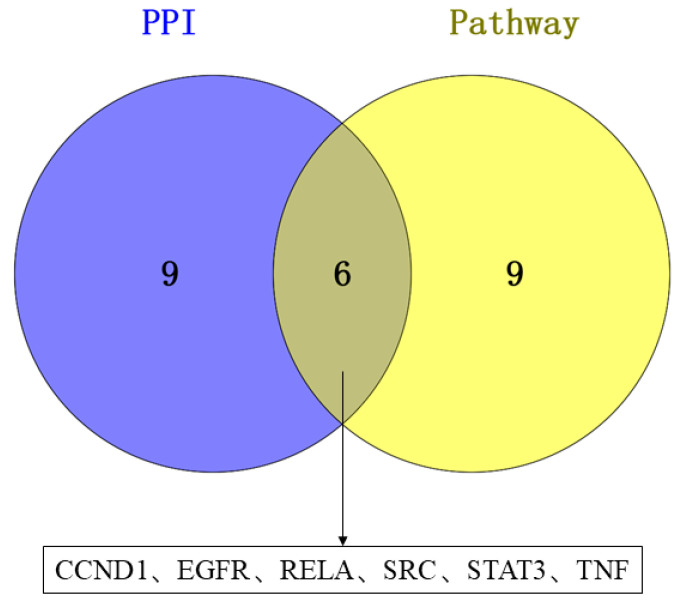
Venn diagram depicting the overlap of core targets identified from PPI analysis and the drug–compound–target–pathway network.

A compound–target–pathway network was established using Cytoscape 3.10.2, integrating all KEGG pathways along with their related targets and active compounds (Figure 10). The following three essential topological parameters were utilized: Degree, CC, and BC, to identify core targets. As shown in Table 5, the top 15 targets were ranked according to their degree values in the network. A Venn diagram analysis revealed six shared core targets, corresponding to the intersection between the top 15 targets from the PPI network and those previously identified (Figure 11).

### 3.7. Validation and Graphical Representation of Molecular Docking Outcomes

**Figure 12:**
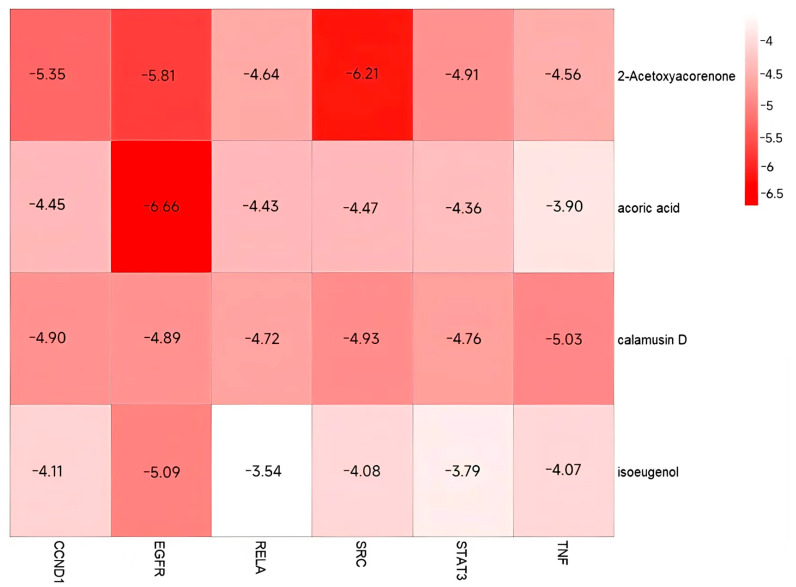
Heatmap of molecular docking scores between core targets and active compounds.

**Figure 13:**
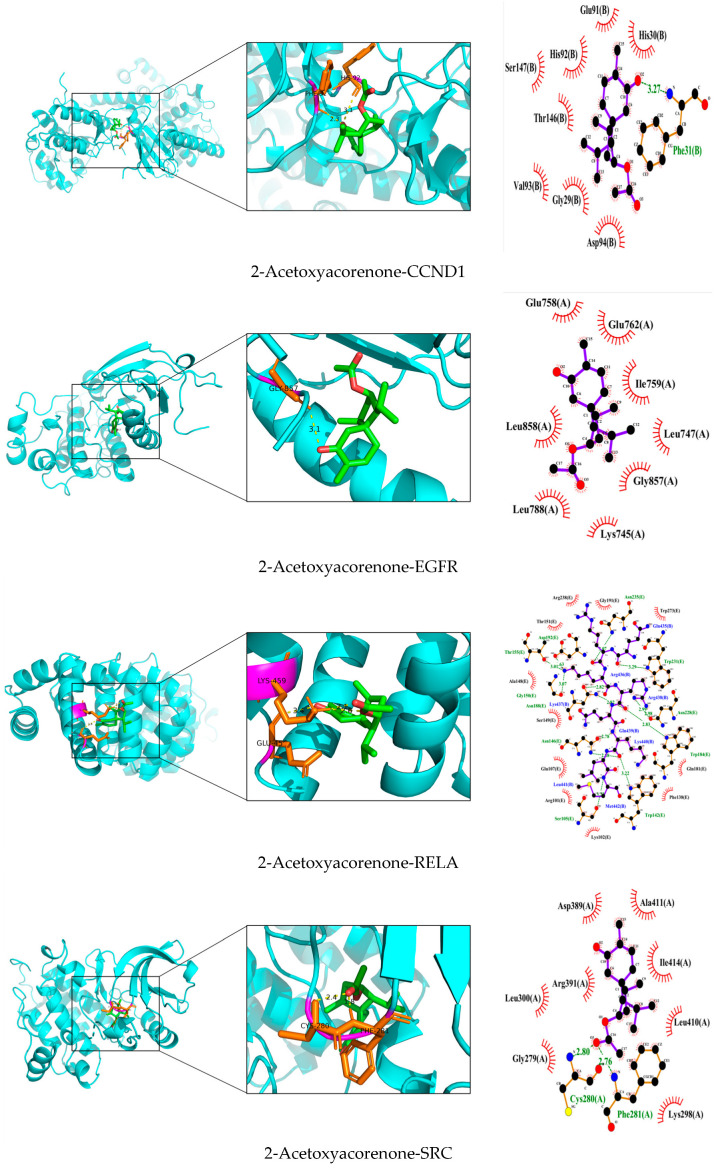

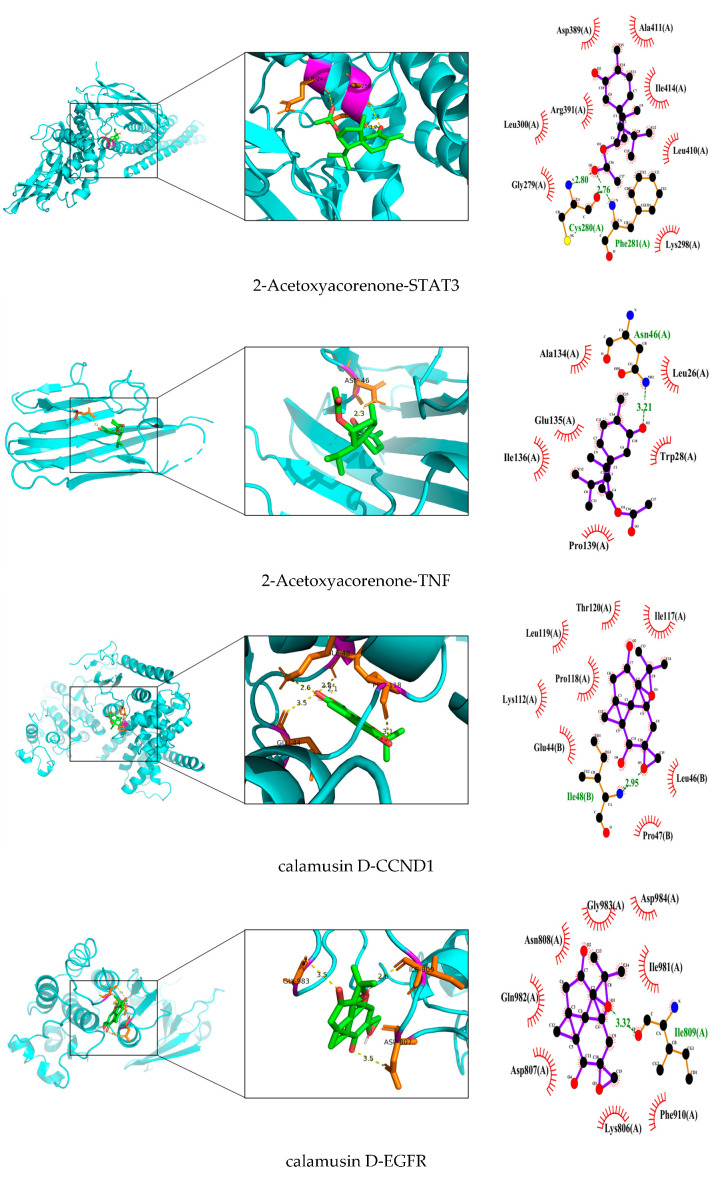

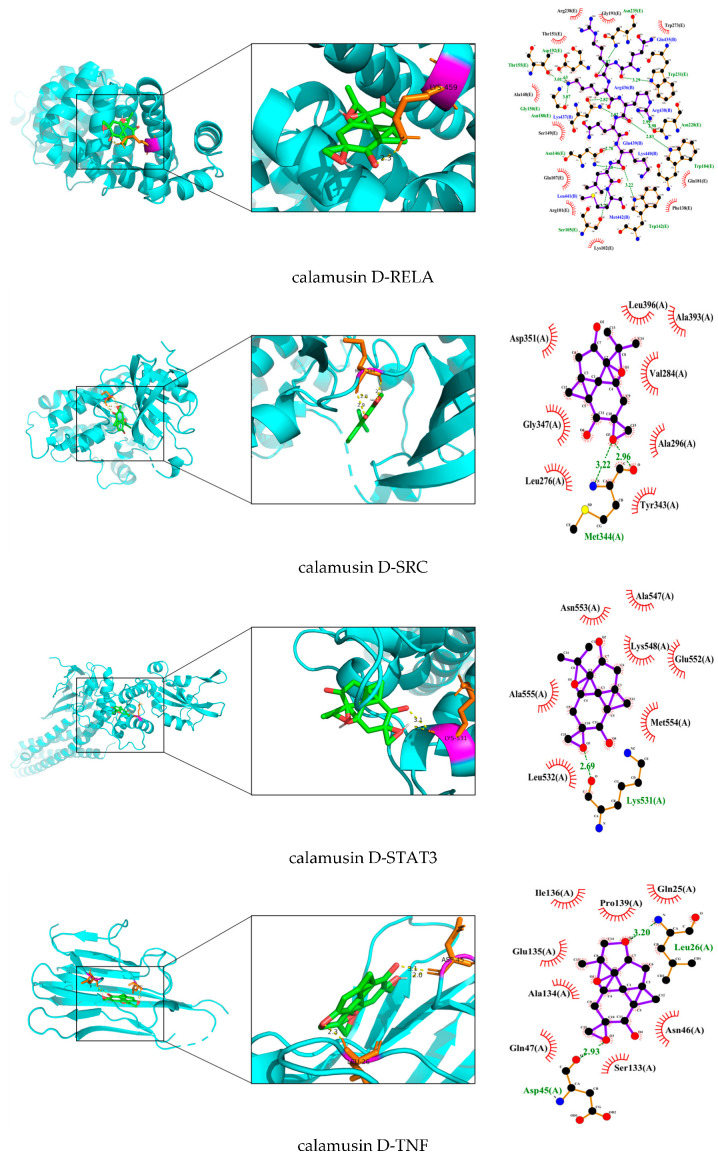

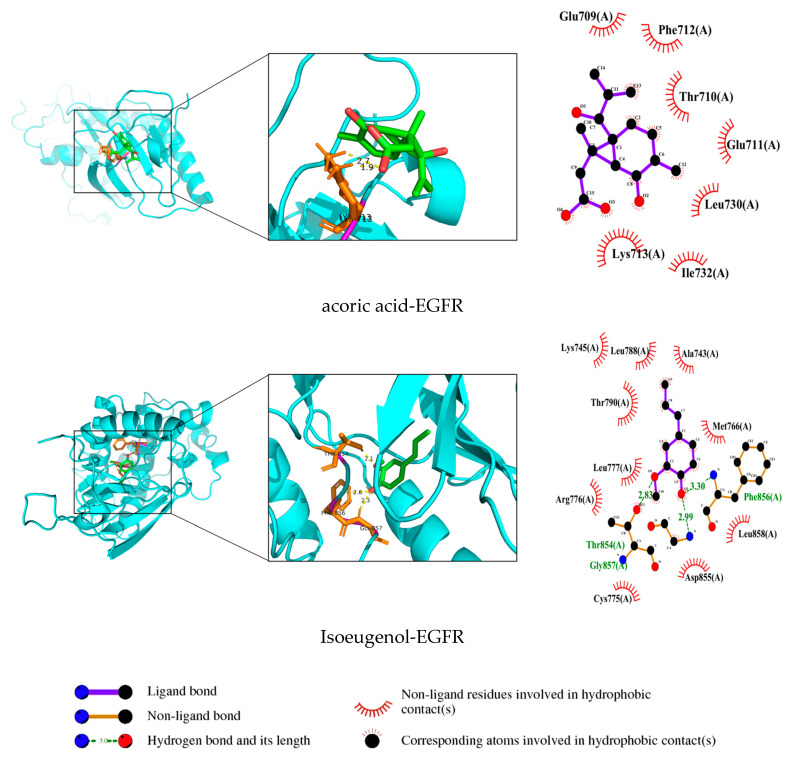
Binding conformations between key targets and bioactive compounds.

To validate the results of the aforementioned analysis, AutoDockTools 1.5.7 was employed to facilitate the molecular docking validation process [21]. This work sought to examine the binding relationships between the selected compound ligands and key target proteins. The molecular docking receptors were retrieved from the UniProt database, with gene names and related PDB IDs summarized in Table 6. The binding energy values, presented as a heatmap (Figure 12), revealed good binding between the target proteins and compounds when the energy was significantly lower than −4.5 kcal/mol. It is evident that CCND1, EGFR, and SRC exhibited lower docking scores with compounds in comparison to other proteins, suggesting that these proteins may serve as prospective binding targets of *Acorus calamus* in the management of RSV. Visualization of the interactions and binding modes of compounds and targets with high free binding energy scores was facilitated by PyMOL and LigPlot (Figure 13). The interactions included hydrogen bonds and hydrophobic interactions, maintaining a stable conformation. The results show that 2-Acetoxyacorenone-SRC interacts similarly with 2-Acetoxyacorenone-STAT3. This may be due to the presence of a region of structural homology between SRC and STAT3, to which 2-Acetoxyacorenone binds. More notably, the interaction of the molecule with RELA is particularly complex. This may be related to the functional diversity of RELA, an important member of the NF-κB family, which is involved in numerous physiological functions and has more corresponding binding sites.

### 3.8. Validation of Core Targets by RSV Datasets from GEO

**Figure 14:**
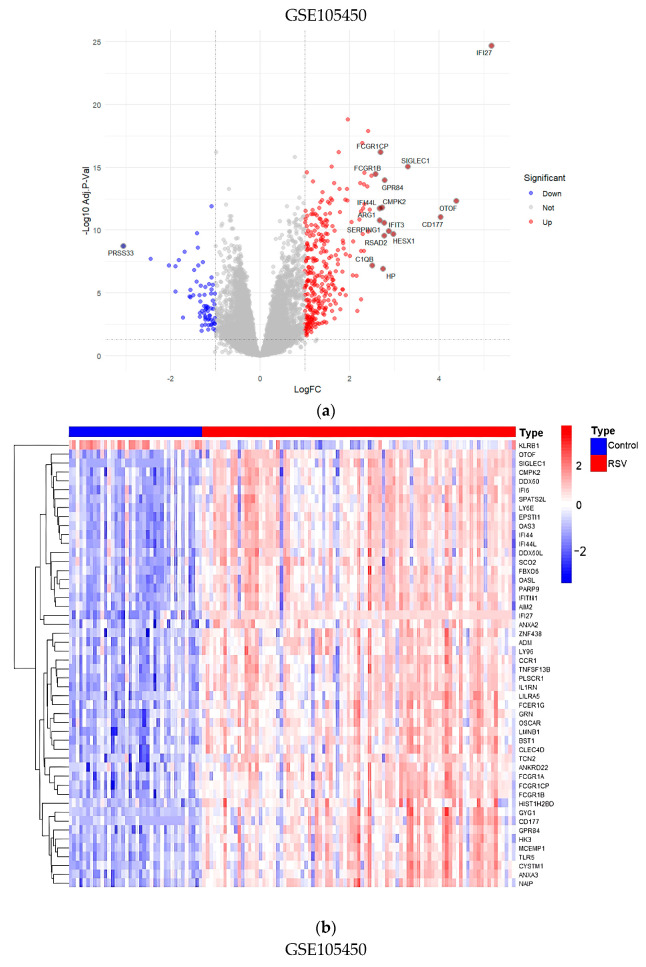

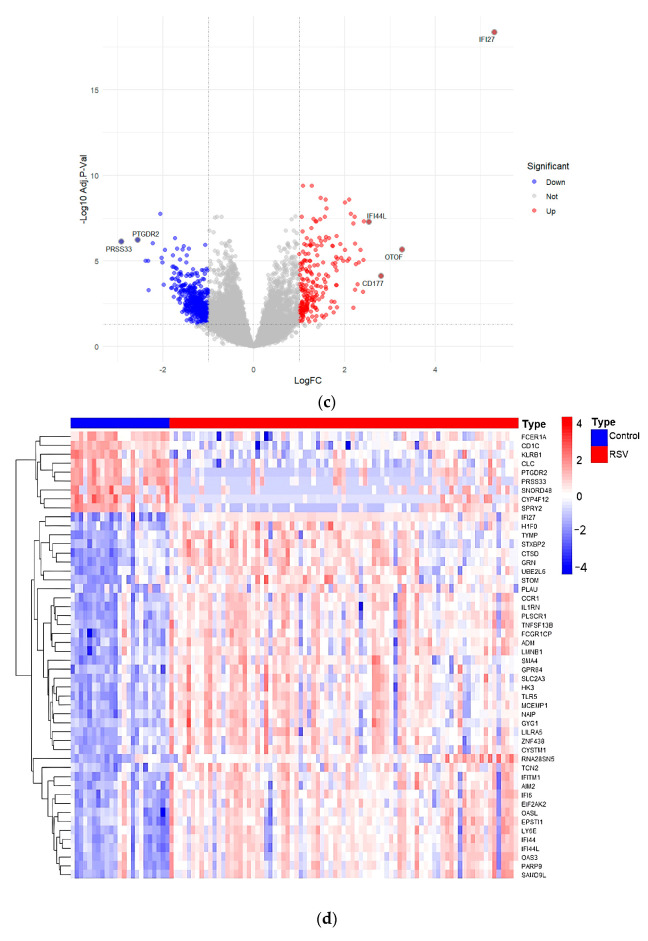

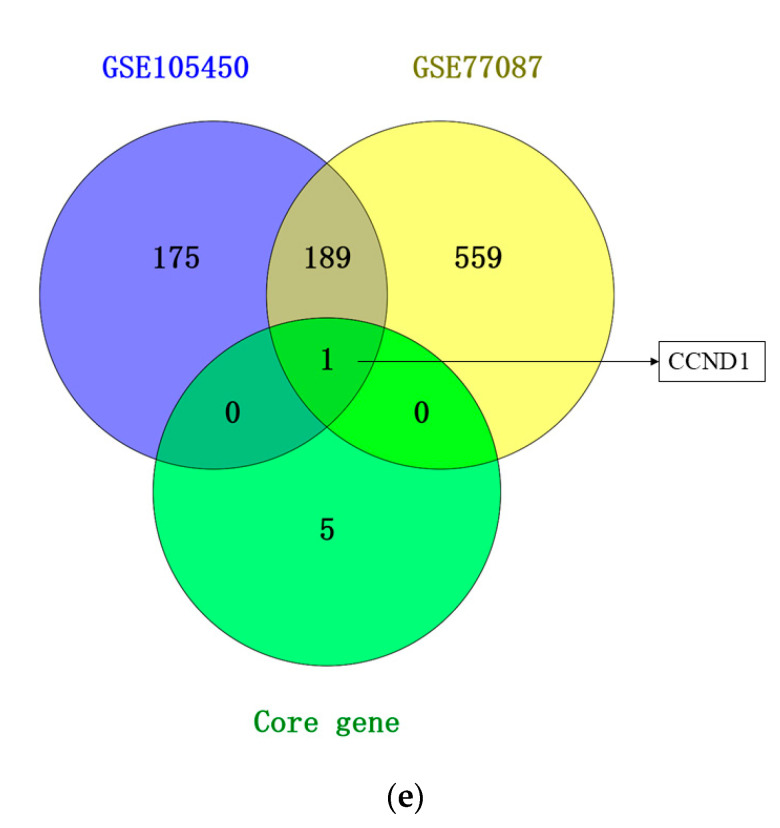
Validation of core targets from datasets of GEO (GSE105450 and GSE77087). (**a**) Volcano plot of DEGs in GSE105450. (**b**) Heatmap of the top 50 DEGs in GSE105450. (**c**) Volcano plot of DEGs in GSE77087. (**d**) Heatmap of the top 50 DEGs in GSE77087. (**e**) Identification of the core gene CCND1.

**Figure 15:**
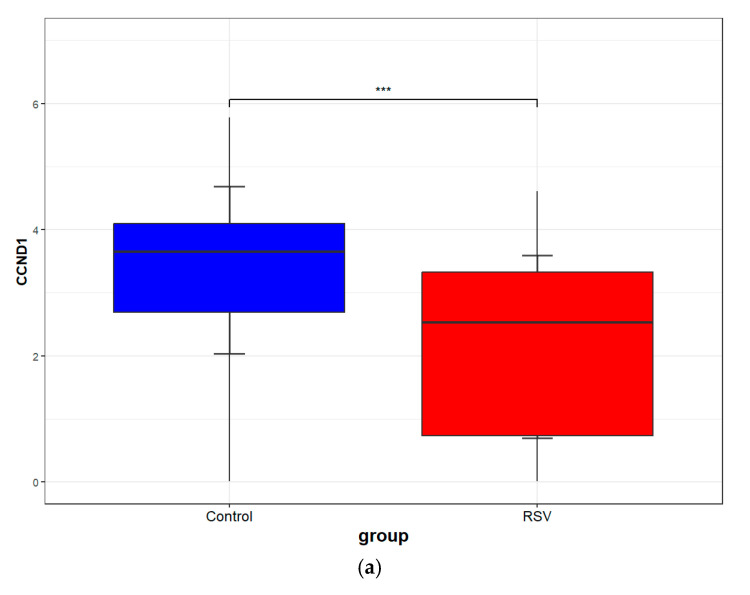

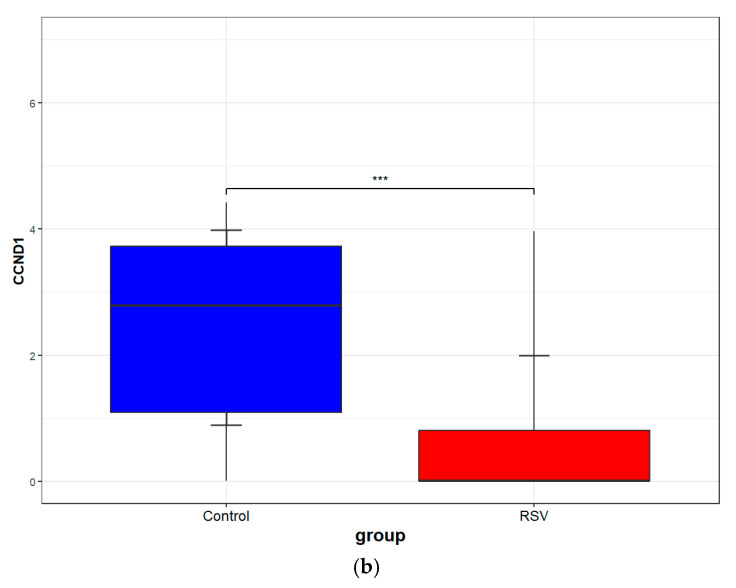
CCND1 expression in the two RSV datasets. (**a**) CCND1 expression of GSE105450. (**b**) CCND1 expression of GSE77087. ***p < 0.001 versus control.

**Figure 16:**
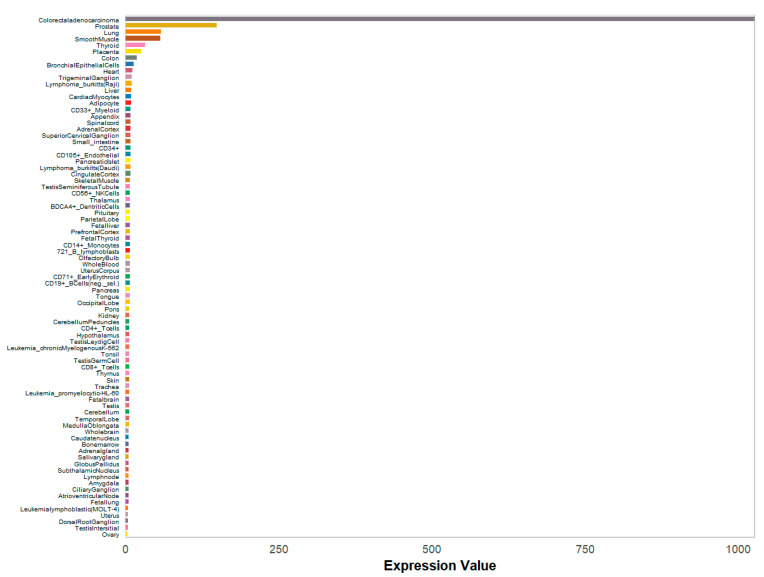
mRNA expression levels of CCND1 in different cells and tissues from BioGPS.

The two RSV datasets, GSE105450 and GSE77087, from the GEO database were subjected to differential analysis to identify potential targets. Volcano plots (Figure 14a,c) illustrate the Differentially Expressed Genes (DEGs), with red dots signifying upregulated genes and blue dots representing downregulated ones. A heatmap was generated to illustrate the distribution of DEGs, with the top 50 further grouped for in-depth analysis (Figure 14b,d). By intersecting the core targets with the two datasets, CCND1 was identified as a common core target (Figure 14e). The CCND1 expressions of the two RSV datasets were visualized separately using box plots (Figure 15). In addition, we assessed the mRNA expression levels of CCND1 in different cells and tissues using data from the Biological Gene Expression Profiling and Annotation-Based Tissue-Oriented Portal database (BioGPS: https://biogps.org) (Figure 16). Consistent with our findings, the results showed that CCND1 expression was higher in lung tissue.

## 4. Discussion

In this study, the existing literature was thoroughly reviewed with the objective of identifying 26 active compounds and 268 associated targets of *Acorus calamus*. In addition, 1633 RSV-related targets were retrieved from the GeneCards and OMIM databases. Eventually, 87 common targets were identified and displayed in a network diagram. During the subsequent DO analysis, a strong association between bronchial disease and RSV infection was revealed, which is consistent with current knowledge. It was discovered that upon RSV infection, viral particles are released and transferred to the fine bronchial tubes of the respiratory tract or alveoli, causing bronchial diseases such as capillary bronchitis [22]. Research has demonstrated that RSV infection can cause abnormalities in human immune regulation and airway remodeling with persistent inflammation. These effects subsequently increase the risk of the development of asthma [23] and induce acute exacerbation of Chronic Obstructive Pulmonary Disease (COPD) [24].

In order to gain further insight into the molecular mechanism of interaction of *Acorus calamus* with RSV infection, a follow-up analysis was performed. GO annotation revealed the biological processes implicated in RSV infection, encompassing chemotaxis, leukocyte migration, regulation of apoptotic signaling pathways [25], and response to oxidative stress [26]. KEGG analysis indicated that the shared targets were predominantly enriched in pathways strongly associated with RSV, such as Leukocyte transendothelial migration [27], C-type lectin receptor signaling pathway [28], and Lipid and atherosclerosis [29]. Notably, within the KEGG analysis results, the majority of targets were centrally concentrated in cellular signaling and regulatory pathways, specifically the PI3K-Akt signaling pathway [30] and the JAK-STAT signaling pathway [31]. The PI3K-Akt signaling pathway has been demonstrated to promote the replication of RSV through the inhibition or delay of apoptosis in airway epithelial cells. Following the infection of respiratory epithelial cells by RSV, the host activates the JAK-STAT signaling pathway through the secretion of type I interferon (IFN) to inhibit viral replication and spread. Nevertheless, the concept of a function for the PI3K-Akt signaling pathway in lung injury remains a subject of debate. It has been shown by some studies that lung inflammation can be alleviated by activation of the PI3K-Akt signaling pathway [32].

Key proteins and compounds were validated through molecular docking to elucidate the therapeutic mechanism of *Acorus calamus* against RSV. Specifically, six RSV-associated core proteins (CCND1, EGFR, RELA, SRC, STAT3, and TNF) were docked with four bioactive molecules (2-Acetoxyacorenone, calamusin D, acoric acid, and isoeugenol) to assess their binding interactions. It becomes evident that CCND1, EGFR, and SRC have lower binding energies and show better binding affinity than other target proteins. Notably, the binding energy of EGFR to all four active molecular compounds was less than −4.5 kcal/mol.

Research has demonstrated that RSV infection significantly downregulates CCND1 expression. In contrast, L-sulforaphane (LSF) can alleviate RSV infection in human lung epithelial cells by upregulating CCND1 levels [33]. RSV, in its early infection stage, activates EGFR-driven survival signaling and directs the mitochondrial translocation of EGFR, thereby prolonging its functional state. The EGFR-mediated signaling cascade plays a critical role in the initial phase of RSV infection [34]. Additionally, Xu’s study [35] revealed that the RELA∙OGG1 complex influences the N-glycosylation modification of RSV glycoproteins. This modification is essential for the RSV-induced adaptive epithelial response and extracellular matrix remodeling, as well as being a key factor contributing to long-term lung developmental abnormalities following early-life RSV infection. Moreover, DTX3L can bind to SRC to mediate TBK1 phosphorylation, which enhances the type I interferon-mediated antiviral response against RSV [36]. Zhao’s research [37] concluded that STAT3-mediated anti-apoptotic signaling protects against severe RSV infection in infants and mitigates both infection severity and inflammation in the lungs of affected children. Other studies have provided evidence that elevated nasal TNF levels are positively associated with the severity of RSV infection, suggesting that TNF upregulation may induce an exaggerated inflammatory response in the airways, thereby worsening RSV infection [38]. Notably, SRC serves as an upstream regulator of EGFR activation and phosphorylation, processes that are integral to RSV infection dynamics [39].

Furthermore, analysis showed that both RSV datasets from the GEO repository shared CCND1, reinforcing its critical involvement in RSV pathogenesis. CCND1 serves as a critical regulator of the G1/S cell cycle transition, which is arrested following RSV infection [40,41]. Additionally, miR-34a has been shown to induce G1 phase arrest by down-regulating CCND1 [42]. Furthermore, RSV G protein-mediated miRNA let-7f can inhibit CCND1 translation [43]. These findings collectively elucidate the mechanism of CCND1 action during RSV infection: down-regulation of CCND1 results in G1/S phase transition arrest of the cell cycle, thereby promoting RSV replication. In addition, researchers have shown that knocking down CCND1 followed by inhibiting the PI3K/AKT/mTOR signaling pathway is effective [44], and that overexpressing CCND1 led to an enhancement in pathway activity [45]. Downregulation of CCND1 after RSV infection might result in the inhibition of the PI3K-Akt signaling pathway. This, in turn, inhibits the anti-inflammatory effects in the lungs that PI3K-Akt activation brings. Activation of the JAK-STAT signaling pathway has been demonstrated to induce a range of physiopathological processes, including cell proliferation, differentiation, and tumorigenesis [46]. These processes may be closely related to CCND1. Nevertheless, there have been no definitive studies conducted thus far to demonstrate a clear correlation between CCND1 and JAK-STAT. Furthermore, evidence has been found for a cross-interaction pattern between various signaling pathways [47]. In conclusion, CCND1 has a complex and close relationship with both the PI3K-Akt and JAK-STAT signaling pathways.

In the domain of RSV treatment, ribavirin was sanctioned by the US Food and Drug Administration (FDA) in 1986 and is the sole pharmaceutical agent authorized to date for the management of RSV infections [48]. Nevertheless, due to its substantial toxic side effects, its use is only of some value in immunodeficient patients and infants with severe RSV infection [49]. Monoclonal antibodies, on the other hand, are an effective option for the treatment of RSV infection. However, only one such antibody, Palivizumab (Synagis^®^), is currently licensed. Palivizumab (Synagis^®^) is effective in the treatment of RSV infections; however, its limited duration of action and the high cost of treatment limit its use [50]. Recently, a new monoclonal antibody, Nirsevimab (Beyfortus^®^), has demonstrated the potential for a more protracted protective effect [51]. Despite these advances, the field still lacks a definitive and highly effective treatment for RSV.

In this study, CCND1 exhibited significant docking activity with 2-Acetoxyacorenone, suggesting the potential efficacy of 2-Acetoxyacorenone as a therapeutic agent for RSV infection. Given the major limitations of existing drugs and methods for RSV treatment, this study proposes a novel approach: targeting CCND1 for the management of RSV infection. In addition, it is worth noting that despite its traditional use as a Tibetan medicinal herb, there has been a lack of in-depth investigation into the mechanisms of disease treatment using *Acorus calamus* and its active compounds. In the case of 2-Acetoxyacorenone, it was initially identified as a sesquiterpene constituent in *Acorus calamus* in earlier studies [52]; however, no further exploration of its bioactive role has been conducted to date. The present study elucidates the potential mechanism by which *Acorus calamus* may act against RSV infection and indicates that 2-Acetoxyacorenone could serve as a promising drug candidate for targeting CCND1 in RSV therapy. This work provides a solid foundation for future research aimed at uncovering the bioactive properties of 2-Acetoxyacorenone.

## 5. Conclusions

Our research conducted a systematic analysis of the molecular mechanisms underlying the interaction of *Acorus calamus* on RSV by integrating network pharmacology and the molecular docking technique. The results demonstrated that 2-Acetoxyacorenone, calamusin D, acoric acid, and isoeugenol might serve as effective components for RSV infection, with CCND1, EGFR, and SRC identified as core genes. Notably, 2-Acetoxyacorenone stood out as the most critical compound, acting as a potential drug candidate targeting CCND1. Furthermore, *Acorus calamus*’s therapeutic action against RSV encompasses various pathways, such as the PI3K-Akt signaling pathway and the JAK-STAT signaling pathway.

In conclusion, the present study revealed that *Acorus calamus* exhibits certain anti-RSV activity and holds promise as an effective drug for treating RSV infection within the Tibetan medicine system, demonstrating significant research and development value. This study has provided a new insight into the molecular basis of *Acorus calamus* in RSV infection and will act as a valuable reference point for future studies in this area.

The present study systematically and comprehensively analyzed the molecular mechanism of *Acorus calamus* in the infection of RSV by combining multiple analytical methods, thus providing a new basis for the treatment of RSV. However, it should be noted that this study is not without its limitations. Firstly, the sources of drug components and targets in this study were obtained from the literature and databases, and the accuracy of these data depends on the quality of the sources. Secondly, the reliability of the results may have been limited by our failure to use a positive control during molecular docking. Thirdly, our study lacks an Absorption, Distribution, Metabolism, Excretion, and Toxicity (ADMET) assessment of *Acorus calamus*, which represents a critical piece of information that should be incorporated in subsequent research. Finally, while this study provides a theoretical basis for the treatment of RSV, further verification of the correctness and practical value of the theory through animal experiments and clinical trials is necessary.

## Figures and Tables

**Table 1:** Details of the 26 active compounds of *Acorus calamus*.

NO.	Compound Name	PubChem CID	Structure
1	2-Acetoxyacorenone	10850234	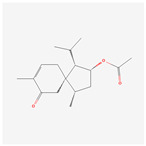
2	calamusin D	60156053	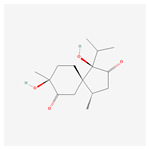
3	acoric acid	15558301	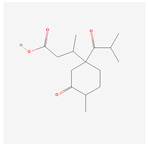
4	isoeugenol	853433	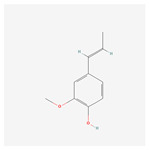
5	Methyl palmitate	8181	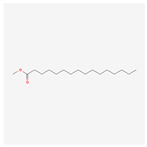
6	eugenol	3314	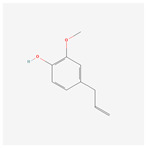
7	(Z)-Methyl isoeugenol	1549045	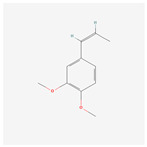
8	methyl isoeugenol	637776	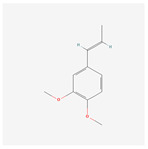
9	Calamensesquiterpinenol	75250012	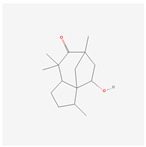
10	Calamusin F	60156148	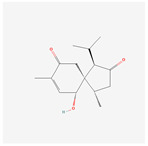
11	camphor	2537	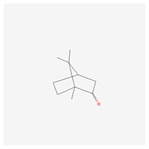
12	Calamusin E	60156054	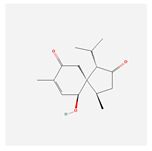
13	eugenyl acetate	7136	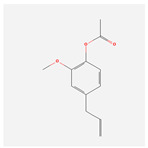
14	bullatantriol	71430886	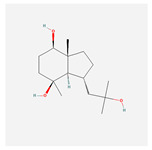
15	2,4,5-Trimethoxybenzoic acid	10276	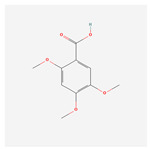
16	Cedranone	111402	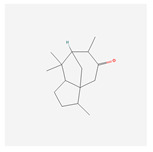
17	Dehydroxy-isocalamendiol	535379	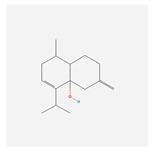
18	* cis * –asarone	636822	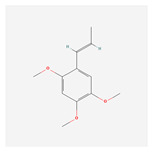
19	Calamusin I	60156151	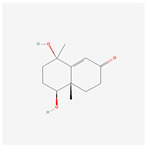
20	Isoacoramone	3083746	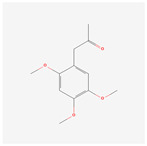
21	β asarone	5281758	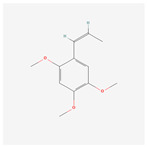
22	acorone	5316254	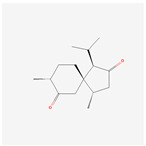
23	tau-Muurolol	6432221	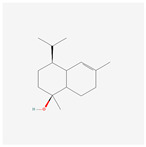
24	(E)-3-(2,4,5-Trimethoxyphenyl)acrylaldehyde	9813266	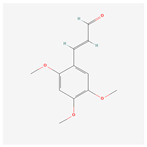
25	oplodiol	12313756	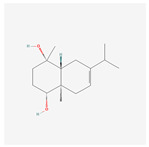
26	Thujopsanone	13893399	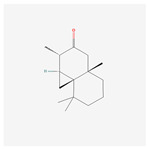

**Table 2:** The 87 overlapping targets shared by active compounds and disease-associated targets.

NO.	Target	NO.	Target	NO.	Target	NO.	Target
1	ABCB1	23	CYP19A1	45	ITGB2	67	PIK3CD
2	ABCG2	24	CYP1A1	46	JAK1	68	PPARA
3	ACE	25	CYP2C9	47	JAK2	69	PPARD
4	ACHE	26	DYRK1A	48	KDR	70	PPP1CA
5	AGTR2	27	EGFR	49	KMO	71	PSMB5
6	AHR	28	EPAS1	50	MAPK14	72	PTGDR2
7	ALOX5	29	EZH2	51	MAPKAPK2	73	PTGES
8	AOC3	30	F2	52	MDM2	74	PTGS2
9	APP	31	F2R	53	MIF	75	PTPN1
10	AR	32	FABP4	54	MMP2	76	PTPN11
11	BRD4	33	FGFR1	55	MPO	77	PTPN2
12	CCNA2	34	FLT1	56	MTNR1A	78	RELA
13	CCND1	35	G6PD	57	MTNR1B	79	RHOA
14	CCR1	36	HDAC6	58	NFE2L2	80	SRC
15	CDK1	37	HMGCR	59	NLRP3	81	STAT3
16	CHRM1	38	HSD11B1	60	NOS2	82	TACR1
17	CNR2	39	HSD11B2	61	NR3C1	83	TNF
18	CREBBP	40	HSPA1A	62	NR3C2	84	TRPV1
19	CTSB	41	ICAM1	63	PABPC1	85	TYK2
20	CTSL	42	ITGAL	64	PARP1	86	VDR
21	CXCL8	43	ITGAV	65	PGR	87	VEGFA
22	CXCR2	44	ITGB1	66	PIK3CB		

**Table 3:** Details of the top 10 compounds in the network between herb compounds and disease targets.

Rank	Compound Name	PubChem CID	Degree
1	2-Acetoxyacorenone	10850234	41
2	calamusin D	60156053	24
3	acoric acid	15558301	22
4	isoeugenol	853433	21
5	Methyl palmitate	8181	13
6	eugenol	3314	9
7	(Z)-Methyl isoeugenol	1549045	4
8	methyl isoeugenol	637776	4
9	Calamensesquiterpinenol	75250012	3
10	Calamusin F	60156148	3

**Table 4:** Details of the top 15 core targets from PPI network.

Rank	Gene Name	Score
1	STAT3	1.66 × 10^9^
2	EGFR	1.66 × 10^9^
3	TNF	1.66 × 10^9^
4	CCND1	1.63 × 10^9^
5	PARP1	1.39 × 10^9^
6	MDM2	1.38 × 10^9^
7	EZH2	1.22 × 10^9^
8	AR	1.21 × 10^9^
9	RELA	1.17 × 10^9^
10	CREBBP	1.10 × 10^9^
11	SRC	1.06 × 10^9^
12	CDK1	1.04 × 10^9^
13	HDAC6	1.00 × 10^9^
14	BRD4	5.19 × 10^8^
15	MMP2	5.07 × 10^8^

**Table 5:** Details of the top 15 core targets from compound–target–pathway network.

Rank	Gene Name	Degree	CC	BC
1	PIK3CB	92	0.498998	0.080407
2	PIK3CD	92	0.498998	0.080407
3	RELA	72	0.447038	0.061346
4	MAPK14	57	0.424191	0.031357
5	TNF	55	0.434555	0.045415
6	EGFR	44	0.399679	0.020541
7	SRC	40	0.395866	0.019708
8	CCND1	39	0.375566	0.011924
9	RHOA	34	0.403566	0.016991
10	STAT3	33	0.387247	0.011039
11	CXCL8	32	0.381317	0.013256
12	JAK1	28	0.378995	0.007265
13	JAK2	26	0.377845	0.007811
14	VEGFA	25	0.374436	0.008855
15	PTGS2	21	0.384853	0.011878

**Table 6:** Correspondence between gene names and PDB IDs.

Gene Name	PDB ID
CCND1	6p8e
EGFR	8a27
RELA	7let
SRC	7ng7
STAT3	6njs
TNF	5uui

## Data Availability

The authors confirm that the data supporting the findings of this study are available within this manuscript and the tables.

## Supplementary Materials

The following supporting information can be downloaded at https://www.mdpi.com/article/10.3390/cimb47090695/s1.
